# Cell-Free Production of Pentacyclic Triterpenoid Compound Betulinic Acid from Betulin by the Engineered *Saccharomyces cerevisiae*

**DOI:** 10.3390/molecules22071075

**Published:** 2017-06-27

**Authors:** Jianan Wu, Yongwu Niu, Abdelmoneim Bakur, Hao Li, Qihe Chen

**Affiliations:** Department of Food Science and Nutrition, Zhejiang University, Hangzhou 310058, China; 21613036@zju.edu.cn (J.W.); 11713002@zju.edu.cn (Y.N.); bakur888@yahoo.com (A.B.); lihaoenjoy@126.com (H.L.)

**Keywords:** betulinic acid, betulin, *CYP716A12*, *ATR1*, co-expression

## Abstract

Betulinic acid is a product of plant secondary metabolism which has shown various bioactivities. Several *CYP716A* subfamily genes were recently characterized encoding multifunctional oxidases capable of C-28 oxidation. *CYP716A12* was identified as betulin C-28 oxidase, capable of modifying betulin. This study aimed to induce the transformation of betulin to betulinic acid by co-expressing enzymes *CYP716A12* from *Medicago truncatula* and *ATR1* from *Arabidopsis thaliana* in *Saccharomyces cerevisiae*. The microsome protein extracted from the transgenic yeast successfully catalyzed the transformation of betulin to betulinic acid. We also characterized the optimization of cell fragmentation, protein extraction method, and the conversion conditions. Response surface methodology was implemented, and the optimal yield of betulinic acid reached 18.70%. After optimization, the yield and the conversion rate of betulin were increased by 83.97% and 136.39%, respectively. These results may present insights and strategies for the sustainable production of betulinic acid in multifarious transgenic microbes.

## 1. Introduction

**Figure 1:**
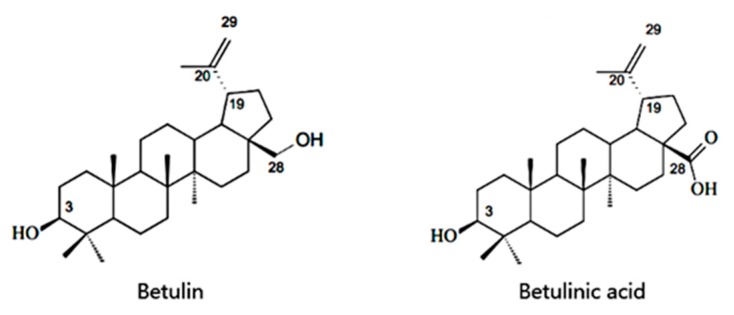
Structures of betulinic acid and betulin.

Triterpenoids are a diverse class of chemical compounds, and they have been increasingly attracting the attention of researchers due to their beneficial bioactivities in the health field. Betulinic acid (3-hydroxy-lup-20(29)-en-28-oic acid) is a pentacyclic lupane-type triterpenoid that is widely distributed throughout the entire plant kingdom. The chemical structures of betulinic acid and its precursor betulin are shown in Figure 1. Recently, among these compounds, betulinic acid has gained considerable interest owing to a variety of biological and pharmacological activities that have been ascribed to this compound including anti-inflammatory, antibacterial, antiviral, antimalarial, anti-HIV, and antitumor effects [1]. Betulinic acid is considered to be a potential prospective anticancer therapeutic agent due to its specific cytotoxicity against cancer cells [2]. Interestingly, despite its extraordinary potential for therapeutic applications, the insufficient sources of betulinic acid in plants is a major challenge in commercializing this therapeutic compound. Though betulinic acid can be extracted from birch bark, which is its most common source, the limited content in bark tissue is still the obstacle for industrial manufacture of betulinic acid to meet market demand [3]. Thus, to develop more methods for preparing this compound is a major challenge. Chemical synthesis based on betulin as the precursor was usually reported. Recently, an alternative approach for producing betulinic acid from betulin was achieved by a biotransformation process, but the conversion efficiency was rather poor, and this metabolic pathway is subject to the restriction of limited betulin supply [4]. Rapid progress in synthetic biology and metabolic engineering provides another way to achieve a high yield of natural products in microbial hosts [5,6,7]. The identification of new microbes that convert betulin to betulinic acid is widely conducted these days, and the metabolic engineering of key biosynthetic genes derived from plant sources is well reported.

The biosynthesis pathway of betulinic acid has been postulated in plants. Two critical steps of it are the synthesis of lupeol from 2,3-oxidosqualene and the subsequent C-28 oxidation by cytochrome P450 (CYP) enzymes. *CYP* genes encoding enzymes capable of C-28 oxidation were characterized. These C-28 oxidases are from plant species including *Medicago truncatula* [8], *Vitis vinifera* [8], *Panax gensing* [9], and *Catharanthus roseus* [10]. Previous research focused on producing betulinic acid from yeast endogenous 2,3-oxidosqualene by combinatory expression of lupeol synthase and lupeol C-28 oxidase. Recently, Zhou et al. [11] isolated a gene encoding lupeol oxidase from *Betula platyphylla* bark (BPLO) and co-expressed BPLO in WAT11 chassis yeast with lupeol synthase. The production of betulinic acid by the Gal80p mutant in the engineered WAT11 yeast reached 0.16 mg/L/OD600 [11]. Biotransformation could be a productive approach to obtain triterpenes by enhancing structural diversity [12]. However, the inhibition or toxicity of betulinic acid or betulin is a major challenge for microbial transformation [13]. It has been reported that betulin induced *Rhodococcus rhodochrous* to form a heterogeneous cell aggregation, which is an adaptive strategy of microorganisms to survive under unfavorable conditions, and the aggregate size was correlated with betulin concentration [14]. Considering the low yield obtained by the engineered yeast or other microbes and its cytotoxicity towards yeast cells, the cell-free system may be a promising alternative for betulinic acid production. Thus, we used the microsome protein extracted from yeast co-expressing *CYP716A12* and *ATR1* to catalyze the biotransformation from betulin to betulinic acid. *ATR1* is a NADPH-CYP reductase from *Arabidopsis thaliana* which serve as a redox partner for *CYP716A12*. After successful construction of the engineered yeast, we aimed to boost the yield of betulinic acid from betulin. Cell disruption and microsome protein extraction methods have important effects on the catalytic activity of microsomes. In addition, NAPDH is vital for this catalysis. Therefore, the cell disruption method, microsome protein preparation method, transformation time, and the concentrations of betulin and nicotinamide adenine dinucleotide phosphate (NADPH) were optimized. The purpose of this work is to construct an engineered yeast co-expressing the two enzymes derived from plant sources to transform betulin into betulinic acid. We also identified the capability of producing betulinic acid from betulin and presented insights and strategies for the sustainable engineering of betulinic acid in multifarious transgenic microbes.

## 2. Results

### 2.1. Target Genes Cloning and the Construction of a Co-Expression System

Both betulin and betulinic acid are pentacyclic lupane-type triterpenoids, the carbon skeletons of these two chemicals are the same. As the C-28 hydroxymethyl of betulin is oxidized to a carboxyl group, it forms betulinic acid. The vital step of transforming betulin into betulinic acid is looking for a multi-functional C-28 oxidase and its corresponding reductase. In a large number of C-28 multi-functional oxidases, *CYP716A12* obtained from the *Leguminosae* plant *Medicago truncatula* was chosen to study the amplification of the *CYP716A12* using total DNA from *Medicago truncatula* leaves by specific primers. ATR1 is a nicotinamide adenine dinucleotide phosphate cytochrome *P450* reductase (NADPH-CYP reductase) existing in *Arabidopsis thaliana*. *ATR1* was amplified by reverse transcriptase using the RNA from seedling leaves of *A. thaliana*. The results of gel electrophoresis of the two genes are presented in Supplementary Figures S1 and S2. The plasmid was successfully constructed and introduced into *E. coli* DH5α for amplification. Then, the pESC-ura-CYP716A12-ATR1 harvested from *E. coli* was introduced into W303-1b yeast and the recombinant strain is designated as *Saccharomyces cerevisiae* ZJUQH311 and stored at CCTCC, No. M 2015662.

### 2.2. Betulin Transformation and Optimization

#### 2.2.1. Microsome Preparation and Betulin Transformation

The microsome concentration gained through grinding and the differential centrifugation was 1.10 ± 0.16 mg microsome/50 mL culture media (*n* = 4). Betulin was converted into betulinic acid in a cell-free system to avoid betulin induced apoptosis and other damage to the yeast cell and, thus, to achieve higher production of betulinic acid.

**Figure 2:**
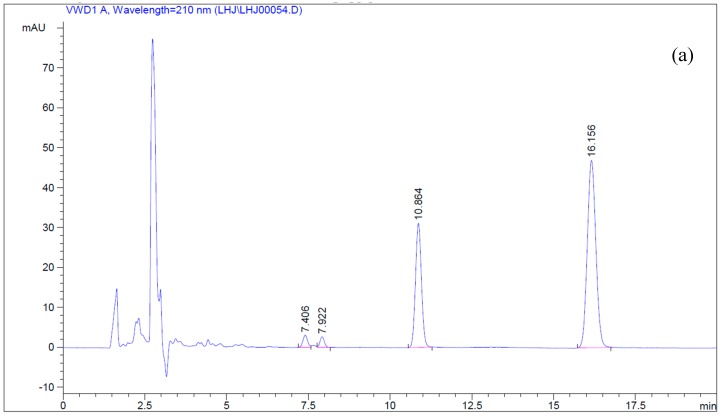

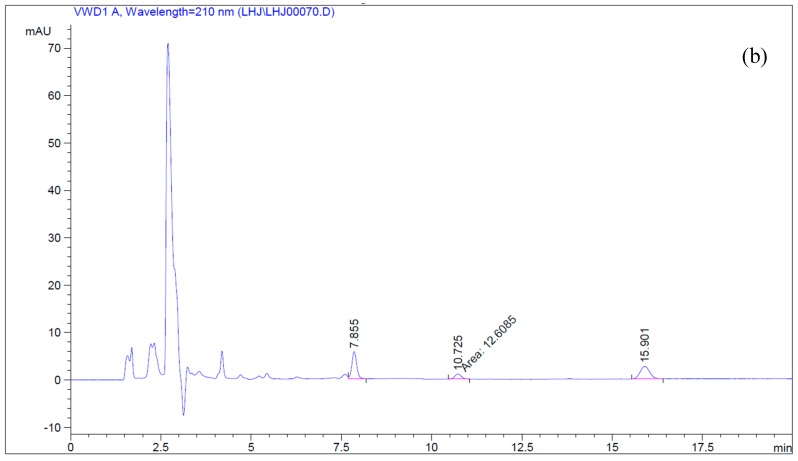
(**a**) HPLC chromatograms of the standards; (**b**) HPLC chromatograms of the biotransformation samples.

The engineered *S. cerevisiae* harboring *CTP716A12* and *ATR1* co-expressed two genes and the microsome harvested was capable of transforming betulin into betulinic acid. The retention times of betulin and betulinic acid are about 16.2 min and 10.9 min (Figure 2a). After biotransformation, betulinic acid was detected and the amount of betulin decreased (Figure 2b). The yield of betulinic acid was 9.67% and the conversion rate of betulin was 19.82%. Liu et al. [4] bred *Armillaria luteo-virens* Sacc ZJUQH by a low energy N^+^-implantation method and isolated a strain with a strong capability of transforming betulin to betulinic acid. After optimization, the predicted optimum yield of betulinic acid by this strain was stable at 9.32% [4]. These results may suggest that the microsome extracted from the engineered yeast has a high efficiency and convenience for betulin-betulinic acid transformation with only 3 h of reaction time, and the yield of betulinic acid was considerable. This verified that the engineered *S. cerevisiae* ZJUQH311 is an effective co-expression system for C-28 oxidation in betulin.

#### 2.2.2. Optimization of Microsome Protein Preparation Methods

**Figure 3:**
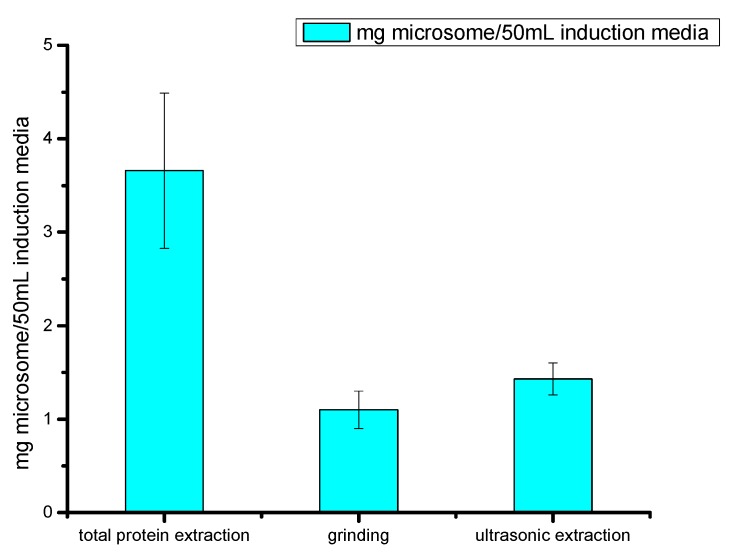
Microsome protein concentration from different protein extraction means.

BCA protein assay kit was used to determine the protein concentration of three microsome protein preparation methods (Figure 3). The total protein extraction was 3.66 ± 0.89 mg microsome protein/50 mL yeast extract peptone dextrose medium with adenine (YPAD) (Gal) (*n* = 4), and the yield of the microsomal protein extraction method was 1.10 ± 0.16 mg microsome protein/50 mL YPAD (Gal) (*n* = 4). Two proteins were compared in transformation capability and the reversed phase HPLC was used to detect betulinic acid and the remaining amount of betulin (Supplementary Figures S12 and S13). Though the concentration of total protein extraction is higher, it seems that the protein cannot transform betulin into betulinic acid. On the other hand, the protein extracted by the microsomal method was efficient in its transformation. The reason for this may be that the mercapto group within the β-mercaptoethanol used in the microsome protein extraction method protected the proteins from oxidation, the d-glucitol balanced the osmotic pressure, and the BSA (bovine serum albumin) protected the protein from denaturation and specific adsorption. This decreased the denaturation, negative effects from chemicals, and the loss because of adsorption to the wall. The microsome protein extraction method has a protective effect on enzymes, but the total protein extraction may cause the oxidation and degradation of these enzymes.

#### 2.2.3. Comparison of Cell Fragmentation Methods

Two normal types of physical fragmentation were chosen to compare with each other since chemical fragmentation is costlier, less efficient, and has a greater chance of contamination. The comparison of fragmentation methods is based on microsome protein extraction (Figure 3). The utilization of bead grinding obtained 1.10 ± 0.16 mg microsome/50 mL YPAD (Gal), and the ultrasonic fragmentation obtained 1.43 ± 0.14 mg microsome/50 mL YPAD (Gal). Under the same culture conditions and the same protein extraction method, ultrasonic fragmentation resulted in higher protein concentration than that of grinding fragmentation (*p* < 0.05). The transformation capability of protein from ultrasonic fragmented cells was confirmed by HPLC so that ultrasonic fragmentation can be used as the preparation method for future experiments.

#### 2.2.4. Optimization of Biotransformation Conditions by *S. cerevisiae* ZJUQH311 Microsome Protein In Vitro

After preliminary experiments, the three most significant factors that affect betulinic acid production, transformation time (X_1_), betulin concentration (X_2_), and NADPH concentration (X_3_), were selected. Using the process of Box-Behnken design, a three-level design leading to 17 sets of experiments with four replicates at the center point was employed to optimize the transformation. The experimental design and the results are demonstrated in Table 1. The ranges of the variables were chosen based on preliminary experiments. Response surface methodology can carry out regression analysis to betulin-betulinic acid transformation. We applied polynomial regression analysis to the data of betulin conversion rate and yielded the following equation:

Y = 34.06 − 8.98X_1_ − 1.18X_2_ + 3.77X_3_ + 18.43X_1_^2^ − 1.77X_2_^2^ − 3.82X_3_^2^ − 0.54X_1_X_2_ + 2.13X_1_X_3_ − 1.72X_2_X_3_ (1)

The coefficient of determination is 0.9260. The variance analysis of the data (Supplementary Table S1) indicated that this model fitted well. The response surface of the equation is showed in Supplementary Figure S14. The predicted optimal conversion rate of betulin reached 61.81% under the combination of X_1_ = 3 h, X_2_ = 71.6 μM, and X_3_ = 1.28 mM.

Polynomial regression was used for the analysis of betulinic acid yield, and the following quadratic polynomial equation was used:

Y = 7.23 + 3.33X_1_ − 3.97X_2_ + 0.43X_3_ − 0.24X_1_^2^ + 1.77X_2_^2^ + 0.81X_3_^2^ − 0.97X_1_X_2_ + 1.64X_1_X_3_ + 1.23X_2_X_3_ (2)

**Figure 4:**
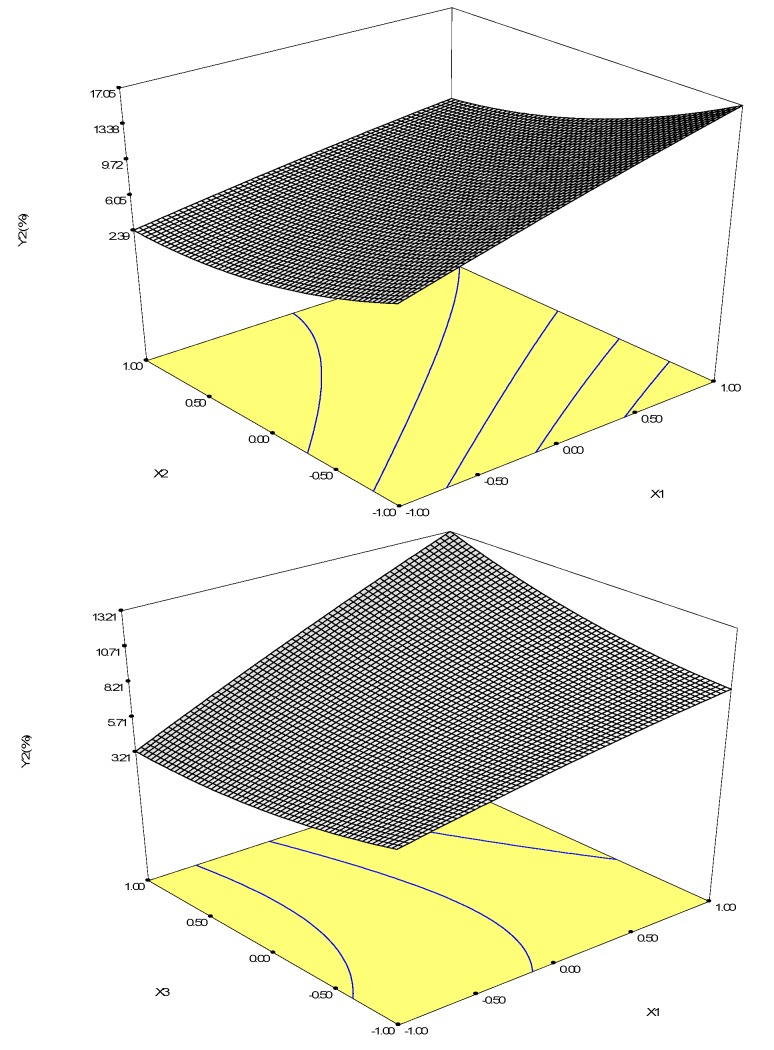

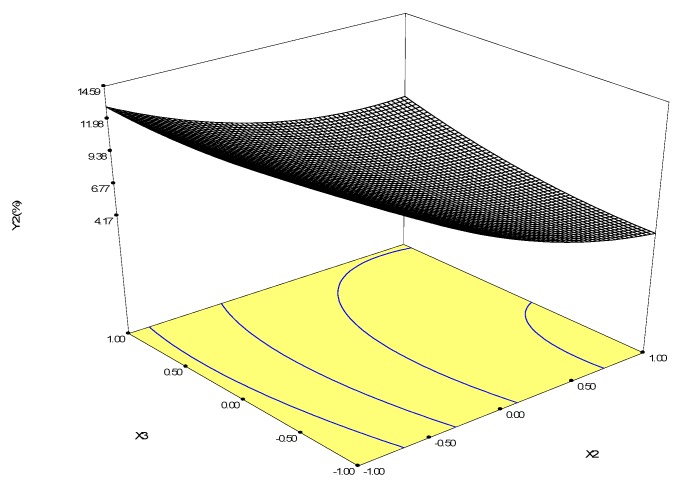
Response surface plot of conversion time (X_1_), betulin (X_2_), and NADPH (X_3_) against betulinic acid yield.

The coefficient of determination is 0.8911. The variance analysis of the data (Table 2) indicated that this model fits well. The visualization of the predicted model equation is displayed by the response surface plots (Figure 4). Because we have three variables in this experiment design, each plot surface was visualized when one variable was set to a constant value. Thus, three surfaces were generated. The surfaces generated can be used to indicate the direction in which the original design must be displaced in order to attain the optimal conditions [15]. For quadratic models, the optimal point can be obtained by calculating the coordinates of the critical point through the first derivate of the mathematical function [15]. In the present study, the calculation was conducted in the statistical software Design Expert 8.0. As we can see from the response surface plots, X_1_ and X_2_ are more significant factors than X_3_. According to the response surface, the predicted yield of betulinic acid reached 18.68% when X_1_ = 9 h, X_2_ = 40 μM, and X_3_ = 1.99 mM. When X_1_ = 9 h, X_2_ = 40 μM, and X_3_ = 2 mM, both the conversion rate and the yield reached the optimal values, the conversion rate of betulin reached 47.27% and the productivity of betulinic acid reached 18.70%. Under corresponding conditions, the relative error between both the actual values and the predicted values is less than 5%.

If the biotransformation time is 9 h, betulin is 40 μM, and NADPH is 2 mM, the conversion rate of betulin and the yield of betulinic acid is synergistically optimized. After optimization, the productivity of betulinic acid and conversion rate of betulin were increased by 83.97% and 136.39%, respectively.

## 3. Discussion

In the present study, we cloned an oxidase gene and a reductase gene encoding enzymes that co-catalyze the conversion of betulin to betulinic acid. Engineering the *Saccharomyces cerevisiae* ZJUQH311 co-expressing *CYP716A12* from *Medicago truncatula* and *ATR1* from *Arabidopsis thaliana* to transform betulin into betulinic acid by the extracted microsome protein, and optimizing the conditions for betulinic acid production, proved to be an effective strategy. Betulinic acid discovered in birch bark and a wide range of other plants shows a variety of biological activities including anti-inflammation, antitumor, anti-HIV, and antibacterial activities [16,17,18,19,20,21,22]. The biosynthesis of betulinic acid can be achieved by a series of enzyme catalysis from squalene, a metabolite of FPP (farnesyl pyrophosphate) coupling [8]. The oxidation of squalene and then the catalysis of lupeol synthase followed by a series of oxidations, including C-28 oxidation by *CYP716A*, can form betulinic acid in chassis yeast [8].

*CYP716A12* belongs to the *CYP716* family in the *CYP85* clan [9]. Nelson et al. [23] identified 246 *CYP* genes in *A. thaliana*. *CYP* plays an important part in the oxidation reactions of synthesizing a variety of plant secondary metabolites, such as terpenoids, fatty acids, and pigments [24]. In order to characterize the C-11 oxidation capability of *CYP88D6* in glycyrrhizin biosynthesis, Seki et al. [25] constructed an engineered yeast expressing β-amyrin synthase, so that β-amyrin could be produced endogenously. *CYP88D6* was co-expressed with cytochrome *P450* reductase (CPR), a redox partner in the recombinant yeast [25]. A previous study showed that the over-expression of CPR is essential for electron transfer to plant or animal heterologous *P450*s expressed in chassis yeast [26]. It has also been reported that *ATR1* supported the oxidation by *CYPs* [27]. Thus, in the present study, we cloned ATR1 from *A. thaliana* and expressed it in *S. cerevisiae* as the redox partner for *CYP716A12*.

C-28 oxidase is involved in the biosynthesis from betulin to betulinic acid. Previous studies have identified some *CYPs* which are capable of C-28 oxidation, including *CYP716A12* from *Medicago truncatula* [8], *CYP716A15* from *Vitis vinifera* [8], *CYP716A52v2* from *Panax ginseng* [9], and *CYP716AL1* from *Catharanthus roseus* [10]. *CYP716A12* is a multifunctional enzyme capable of α-amyrin C-28 oxidation, β-amyrin C-28 oxidation, and lupeol C-28 oxidation. The grapes *CYP716A15* and *CYP716A17* are highly expressed in plant stems and fruit skins, catalyze the oxidation of β-amyrin to produce oleanolic acid, and in the transgenic yeast co-expressing α-amyrin synthase (aAs), *CPR*, and *CYP716A15*, betulin and betulinic acid was synthesized and detected [8]. *CYP716A52v2* was reported to be 73% identical to *CYP716A12* from *Medicago truncatula* and when both *CYP716A52v2* and *Panax ginseng* β-amyrin synthase (PNY1) were expressed in yeast, the transformation from β-amyrin to oleanolic acid was also detected. Similarly, *CYP716AL1* from *C. roseus* co-expressed with lupeol synthase from *A. thaliana* in yeast cells also showed the ability to produce betulinic acid [10].

Herein, the optimization condition is performed by optimizing the reaction time, the substrate concentration, and the supplementation of NADPH. In transgenic yeast producing betulinic acid in vivo, the over-expression of the Glu221Ser/Ile222Arg/Ala223Ser mutant of 2,3-butanediol dehydrogenase with a reasonable range of acetoin supplementation could increase the level of NADPH inside transgenic yeast [28,29,30,31]. The oxygen supply is also essential [30]. The expression of yeast codon optimized *Vitreoscilla* hemoglobin in yeast co-expressing *CYP716AL1* from *C. roseus* and lupeol synthase from *A. thaliana* improved betulinic acid biosynthesis [30]. However, we are still facing some obstacles in manufacturing betulinic acid by engineering microbes, such as the poor yield of betulinic acid, the high cost, and low utilization rate of betulin.

**Figure 5:**
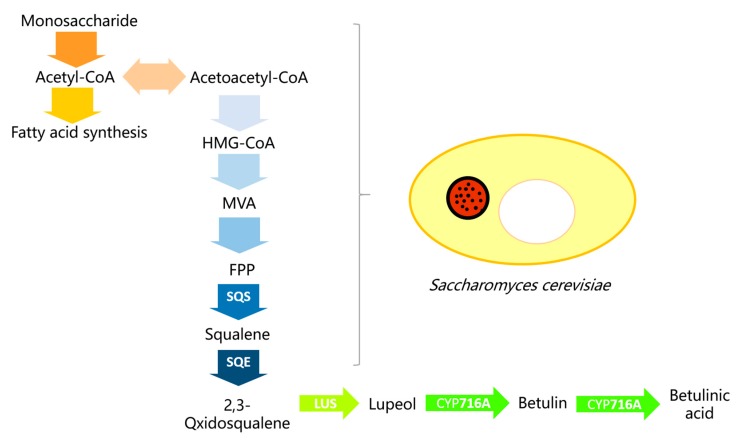
Betulinic acid synthesis pathway. HMG-CoA, hydroxy methylglutaryl coenzyme A; MVA, mevalonic acid; FPP, farnesyl pyrophosphate; SQS, squalene synthase; SQE, squalene epoxidase; LUS, lupeol synthase.

The common culture system for microorganisms is a cold aqueous system. However, the betulin used in previous studies as the substrate is only slightly soluble in it, and the microbial cell wall properties circumscribe the cell uptake of betulin [14]. The utilization of protein transformation in vitro also faces the high cost of betulin and its low conversion rate. The biosynthesis pathway of betulinic acid is postulated in Figure 5. Monosaccharides, through the mevalonate pathway, transform into farnesyl pyrophosphate, then, under the catalysis of relevant synthase and oxidase, lupeol was generated. Finally, the *CYP716A* multifunctional C28-oxidase oxidizes lupeol into betulinic acid [5,8]. The steps before lupeol synthase are endogenous in yeast, so many reports, including the papers we discussed above, introduced lupeol synthase and CYP enzymes into *S. cerevisiae* for betulinic acid production. However, the metabolism of monosaccharides generates acetyl-CoA, and acetyl-CoA is involved in both fatty acid biosynthesis and acetoacetyl coenzyme A production, which limits the substrate source and further results in low productivity. Down regulation of key genes regulating fatty acid synthesis from acetyl coenzyme A or boosting the transcriptions of betulinic acid pathway genes, without causing metabolic disorders or apoptosis in *S. cerevisiae*, may induce the acetyl-CoA flow into the betulinic production pathway and the accumulation of betulinic acid could be greater. When synthetic pathways are constructed under the control of galactose-inducible promotors in *S. cerevisiae*, the activation of *GAL* genes plays an important role in the production of the target compound [26]. A protein complex of Gal3p, Gal4p, and Gal80p controls the induction of *GAL* genes [32]. A recent study isolated a lupeol oxidase gene from *Betula platyphylla* bark [11]. A novel lupeol oxidase was co-expressed with lupeol synthase in WAT11 yeast which contains reductase ATR1. The engineered WAT11 yeast produced betulin and betulinic acid and the Gal80p mutation based on the WAT11 strain significantly increased betulinic acid production [11]. However, the cytotoxicity of betulinic acid is still an obstacle to higher production. Thus, the cell-free system we report here can be a good choice for achieving great productivity. Moreover, breeding and selecting superior betulin-tolerant yeast strains could also be a promising way to achieve higher betulinic acid production. Individual microbial populations are often employed to complete the biosynthesis process of important compounds. However, it has several limitations such as metabolic load, cytotoxicity of its metabolic product to the host, and inability to perform complex tasks. Consequently, by selecting an appropriate mixed community of microbes, it may be possible to build a metabolic pathway from monosaccharide to betulinic acid. Previous studies focusing on using recombinant yeasts to produce betulinic acid endogenously have several advantages including superior substrate accessibility and no need for enzyme extraction. However, low productivity and cytotoxicity of betulinic acid against yeast cells are the major problems. In this study, we provided a cell-free system using microsome protein for betulinic acid production and this system has a high yield of the target product.

## 4. Materials and Methods

### 4.1. Full-Length cDNA Amplification

*CYP716A12* from *Medicago truncatula* and *Arabidopsis thaliana ATR1* were cloned. Gene-specific primers (presented in Table 3) were designed for each sequence. In order to acquire the full length sequence of *CYP716A12* (GenBank: DQ335781.1), the primers C-F and C-R were used to amplify *CYP716A12* with the total DNA from *Medicago truncatula* (kindly donated by Dr. Wang) as the template. The temperature cycles were at 95 °C for 3 min, 95 °C for 10 s, 56 °C for 10 s followed by 40 s at 72 °C (35 cycles) and ending with 10 min at 72 °C. To obtain the full-length *ATR1* sequence (GenBank: NM_118585.3), RT-PCR was conducted using PrimeScript One Step RT-PCR Kit Ver.2 (Takara Bio Inc., Kusatsu, Japan) and RNA isolated from *Arabidopsis thaliana* young leaves. Primers A-F and A-R were used to amplify the ATR1 enzyme. The temperature cycles were 30 min at 50 °C for the reverse transcription, 2 min at 94 °C, 30 s at 94 °C, 30 s at 67 °C, followed by 2 min at 72 °C (30 cycles) and ending with 10 min at 72 °C. The sequence data of the two genes were analyzed by basic local alignment search tool (BLAST) to confirm that the target genes were correctly cloned.

### 4.2. Experimental Strains and Plasmids

Experiments were carried out in *S. cerevisiae* W303-1b (Matα can 1-100 ade 2-1 ura 3-1 trp 1-1 his 3-11, 15 leu 2-3 112). To construct the plasmid pESC-ura-CYP716A12, the insert amplified by PCR using genomic DNA of *CYP716A12* as the template, was ligated into the vector pESC-ura (Agilent Technologies, SantaClara, CA, USA) by homologous recombination using ClonExpress One Step Cloning Kit (Vazyme Biotech, Nanjing, China). Briefly, the vector pESC-ura was linearized at the cloning site using BamHI and NheI restriction enzymes and the end sequence of the linearized vector, a 15 bp homologous region, was introduced into the 5′ end of the primer used for amplifying the *CYP716A12* gene. The 5′ and 3′ ends of the inserted fragments’ PCR product were completely identical to the corresponding ends of the linearized cloning vector. Then, the gel electrophoresis was performed (results shown in Supplementary Figure S5), and the concentration of the PCR product was measured. The recombinant gene was introduced into the competent *E. coli* DH5α, and the *E. coli* was spread on lysogeny broth (LB) plates with ampicillin, then incubated at 37 °C. The positive colonies were selected to make colony PCR and the length of the product was the same with the target gene (Supplementary Figure S6). For further identification, positive colonies were picked for submerged culture and the plasmids extracted were subjected to electrophoresis (Supplementary Figure S7) and PCR sequencing by specific primers. The plasmid pESC-ura-CYP716A12-ATR1 was constructed by ClonExpress One Step Cloning Kit (Vazyme Biotech, Nanjing, China), with ATR1 as the template. In brief, the vector pESC-ura-CYP716A12 was linearized at the cloning site using Bg1II and NotI restriction enzymes (Supplementary Figure S8). The construction process is consistent with the protocol used for pESC-ura-CYP716A12. The successfully constructed pESC-ura-CYP716A12-ATR1 was introduced into *Saccharomyces cerevisiae* by the lithium acetate method. The constructed strain was spread on synthetic complete media lacking uracil (SC-ura) to select positive colonies which were flat and round, and PCR analysis was conducted to confirm the results. The primers and restriction enzymes of the recombinant plasmids are listed in Table 3.

### 4.3. Yeast Expression and Microsome Preparation

*S. cerevisiae ZJUQH311* was used for this expression, which was engineered to achieve the co-expression of the isoform P450 oxidase *Medicago truncatula CYP716A12* and *P450* reductase isoform *Arabidopsis thaliana* ATR1 when induced by adding galactose. Yeast cell proliferation and microsome protein preparation were done as described by Olsen et al. [33] with some modifications. Culturing suspension (100 μL), stored at −80 °C, was inoculated into 10 mL liquid SC minimal medium without uracil and cultured at 30 °C and 180 rpm for 24 h. Then, 50 mL of YPAD (Gal) medium was inoculated by the previous culture at 2 × 10^6^ spores/mL and cultivated at 30 °C and 200 rpm for 12 h. The culture broth was centrifuged (3000× *g*, 5 min) to obtain yeast cells, washed twice with normal saline, and resuspended in YPAD (Gal) medium at 1:15–1:25 (OD_600_ = 2.0) of the inoculation density. Then, it was incubated at 16 °C for 24 h in order for the induction of the microsomes. The following steps were carried out to isolate the microsomes. We centrifuged the yeast culture (2000× *g*, 10 min) and then gently resuspended it using a pipette in 50 mL TEK (100 mM KCl in 50 mM Tris-HCl with 1 mM ethylenediaminetetraacetic acid (EDTA)). After centrifugation (6100× *g*, 3 min), 10 mL extraction buffer (20 mM β-mercaptethanol, 1% BSA, and 0.6 M sorbitol in 50 mM Tris-HCl with 1 mM EDTA) was used for the pipette resuspension. The suspension was then shaken for 49 s × 3 at a vibration frequency of 65 Hz by an automatic shaker. Before another shaking cycle, the suspension was cooled in an ice bath for 5 min. The supernatant obtained from 15 min centrifugation at 6100× *g* was filtered, and to precipitate the microsome protein, MgCl_2_ was added to a final concentration of 50 mM. Before centrifugation at 10,000× *g* for 1 h, the suspension was placed in an ice bath for approximately 1 h. The pellets were dissolved in 1.0 mL to 1.5 mL TEG (30% glycerol in 50 mM Tris-HCl with 1 mM EDTA) and a Teflon pestle was used to homogenize the solution. All experimental steps were carried out on ice; all solutions used were kept at 4 °C.

### 4.4. Transformation Procedure and the Isolation of the Main Metabolites

The transformation system was similar to that previously reported by Mizutani et al. [34]. The experimental procedure was carried out in a total volume of 500 μL of 100 mM sodium phosphate buffer (pH 7.5) containing 1 mM NADPH, 80 μM of betulin, and 1 mg of microsomal fraction protein. The reaction mixture was incubated for 3 h at 30 °C and then extracted with the same volume of ethyl acetate. The aqueous mixture with ethyl acetate was vortex agitated for 1 min and treated with ultrasound for 30 min. The ethyl acetate layer was taken out and blow-dried. The residues dissolved in methanol were used for the analysis by RP-HPLC.

### 4.5. Simultaneous Determination of Betulin and Betulinic Acid by Reverse-Phase HPLC

Betulin and betulinic acid were dissolved in methanol at 2 mg/mL and 1 mg/mL, respectively, in order to prepare the stock solutions. A series of dilutions according to the required concentrations were carried out to prepare the standard solutions for RP-HPLC assay. Standard curves of betulin and betulinic acid were prepared with five different concentrations each. The sample solution was filtered through a 0.22 μm membrane filter, and then an aliquot (10 μL) of the clean solution was injected into the RP-HPLC system. The assaying system used is described in the literature by Liu et al. [4]. In brief, two Waters 510 pumps (Waters, Milford, MA, USA), a sample injector (Rheodyne, Cotati, CA, USA) with a 20 μL loop, and a Waters 996 photodiode array detector made up the RP-HPLC system. A reversed-phase Symmetry C18 (250 mm × 4.6 mm i.d., 4 μL; Waters) column was used with acetonitrile:water in the ratio 91:9 (*v*/*v*) as the mobile phase at a flow rate of 1.0 mL/min under 30 °C. The detection wavelength was set at 210 nm.

### 4.6. Comparison of Different Cell Disruption Methods

Two typical cell disruption methods, grinding fragmentation and ultrasonic crushing, were chosen for the comparative experiments. The grinding fragmentation procedure was the same as mentioned before. For the ultrasonic fragmentation, after the extraction buffer was added, the tubes were placed on ice, and the mixture was treated with ultrasound for 1 min and then paused for 1 min. The experimental steps mentioned above were repeated five times.

### 4.7. Response Surface Methodology Design

In order to optimize the catalytic conditions rapidly, a three-factor Box-Behnken design with three coded levels was performed. Box-Behnken design is one of the response surface methodology experimental designs which is based on three-level partial factorial designs, and its experimental points are located on a hypersphere equidistant from the central point [35]. In this design, the coded values of variables were obtained according to the following equation:

X_i_ = (x_i_ − x_0_)/∆x_i_ (3)

where X_i_ is the coded value of an independent variable, x_i_ is the real value of an independent variable, x_0_ is the real value of an independent variable at the center point, and ∆x_i_ is the step change value. The real values and the coded values of the three variables are showed in Table 1. The quadratic model for predicting the optimal point is expressed according to following equation:

Y = b_0_ + ∑b_i_X_i_ + ∑b_ii_X_i_^2^ + ∑b_ij_X_i_X_j_ (4)

where Y is the response variable, b_0_, b_i_, b_ii_, and b_ij_ are the regression coefficient variables for intercept, linear, quadratic, and interaction terms, respectively, and X_i_ and X_j_ are independent variables. Using the statistical software Design Expert 8.0 to perform the regression analysis, the significance of the regression coefficient was checked by *t*-test, and the significance of the mathematical model equation was then evaluated. The fitting of the equation was determined by R^2^, the coefficient of determination.

### 4.8. Statistical Analysis

All experiments were carried out in triplicate. Design Expert Version 8.0 (Stat-Ease Inc., Minneapolis, MN, USA) was used for performing data-analyses. The treatment effect was evaluated using analysis of variance and Duncan multiple-range test. Differences were considered to be significant at *p* < 0.05 throughout the present study.

## 5. Conclusions

In summary, *CYP716A12* from *Medicago truncatula* and *ATR1* from *Arabidopsis thaliana* were introduced into and co-expressed in *Saccharomyces cerevisiae*. The microsome protein preparation method and ultrasonic fragmentation were shown to be effective at obtaining microsomes. The microsome protein that was extracted was tested for its bio-transforming effects, and the conditions for catalyzing betulin into betulinic acid was optimized by response surface methodology which proved to be a powerful tool. As is clearly shown in the results and Supplementary information, time and betulin concentration are the two most significant factors in the production of betulinic acid. After optimization, the betulinic acid production and betulin conversion rate were increased by 83.97% and 136.39%, respectively. The optimal productivity occurs when the transformation time is 9 h, at which point the concentration of betulin and NADPH are 40 μM and 2 mM, respectively. Further investigations should focus on increasing the microsome production and finding other biochemical pathways that produce betulinic acid by yeast.

## Figures and Tables

**Table 1:** Design and its results for the surface methodology experiment.

Runs	X_1_	X_2_	X_3_	Y_1_ (Betulin Conversion Rate, %)	Y_2_ (Betulinic Acid Yield, %)
1	0	1	−1	21.58	3.33
2	−1	0	−1	61.76	4.14
3	0	−1	−1	20.06	16.93
4	0	0	0	30.28	6.33
5	1	0	−1	35.87	9.10
6	−1	1	0	57.02	4.81
7	−1	−1	0	58.74	7.63
8	0	1	1	33.48	5.16
9	1	−1	0	45.52	14.67
10	−1	0	1	57.25	3.23
11	0	−1	1	38.83	13.83
12	1	1	0	41.66	7.96
13	0	0	0	37.10	7.87
14	0	0	0	32.05	7.56
15	0	0	0	36.89	7.06
16	0	0	0	35.12	7.32
17	1	0	1	39.87	14.75

X_1_: transformation time (h); X_2_: the concentration of betulin (μM); X_3_: the concentration of nicotinamide adenine dinucleotide phosphate (NADPH) (mM). X_1_ = (x_1_ − 6)/3, X_2_ = (x_2_ − 80)/40, X_3_ = (x_3_ − 1)/1.

**Table 2:** Results of the response surface methodology regression analysis for betulinic acid production (Y_2_).

Source	Sum of Squares	DF	Mean Square	F Value	Prob > F
Model	254.21	9	28.25	6.36	0.0117
X_1_	88.97	1	88.97	20.05	0.0029
X_2_	126.38	1	126.38	28.48	0.0011
X_3_	1.50	1	1.50	0.34	0.5793
X_1_^2^	0.23	1	0.23	0.05	0.8247
X_2_^2^	13.25	1	13.25	2.99	0.1276
X_3_^2^	2.76	1	2.76	0.62	0.4564
X_1_X_2_	3.80	1	3.80	0.86	0.3856
X_1_X_3_	10.76	1	10.76	2.42	0.1634
X_2_X_3_	6.07	1	6.07	1.37	0.2805
Residual	31.07	7	4.44		
Lack of Fit	29.71	3	9.90	29.18	0.0035
Pure error	1.36	4	0.34		
Cor Total	285.27	16			

**Table 3:** Primer sequences and restriction enzymes used in this study.

Name	Sequence (5′-3′)	Restriction Enzyme
C-F	AGGAGAAAAAACCCCGGATCCATGGAGCCTAATTTCTATCTCTCCCT	BamHI
C-R	TTAGAGCGGATCTTAGCTAGCTTAAGCTTTGTGTGGATAAAGGCGA	NheI
A-F	AACCCTCACTAAAGGGCGGCCGCATGACTTCTGCTTTGTATGCTTCC	NotI
A-R	GTTAATTAAGAGCTCAGATCTTCACCAGACATCTCTGAGGTATC	BglII
GAL1-F	GGTAATTAATCAGCGAAGCGATG	
GAL1-R	CGAGTCAGTGAGCGAGGAA	
GAL10-F	GGTGGTAATGCCATGTAATATG	
GAL1-R	GGCAAGGTAGACAAGCCGACAAC	

## Supplementary Materials

Supplementary materials are available online.
