# Supplementary material for: Cell-Free Production of Pentacyclic Triterpenoid Compound Betulinic Acid from Betulin by the Engineered Saccharomyces cerevisiae
Source: Molecules. 2017 Jun 27;22(7):1075. doi: 10.3390/molecules22071075 (PMC6152010; doi:10.3390/molecules22071075)
Supplement: Supplementary file 1 [file molecules-22-01075-s001.pdf]

molecules

MDPI

Supplementary Materials (Figures and Tables)

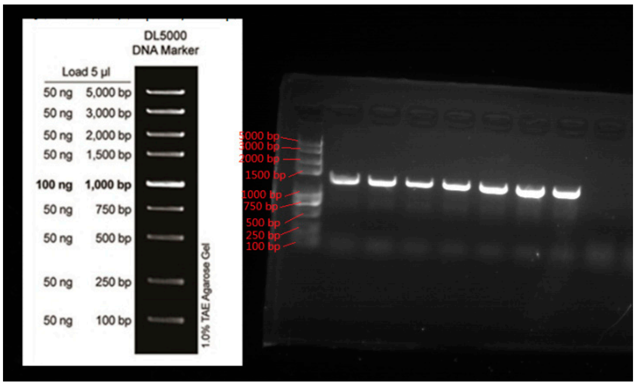

Figure S1. Agarose gel electrophoresis results of CYP716A12.

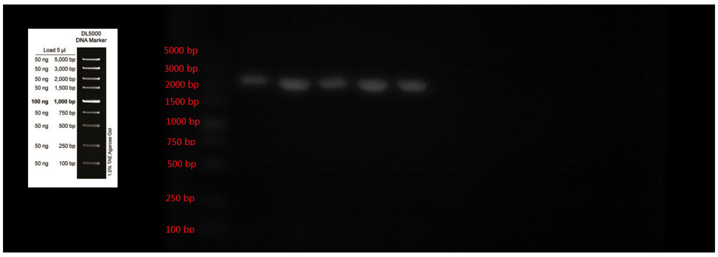

Figure S2. Agarose gel electrophoresis results of ATR1.

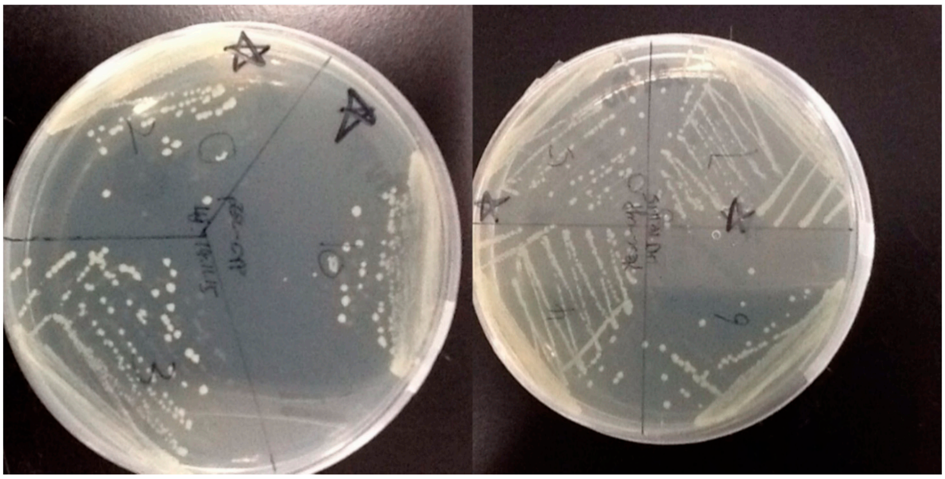

Figure S3. Purification and separation of a pESC-ura-CYP716A12 DH5α single colony.

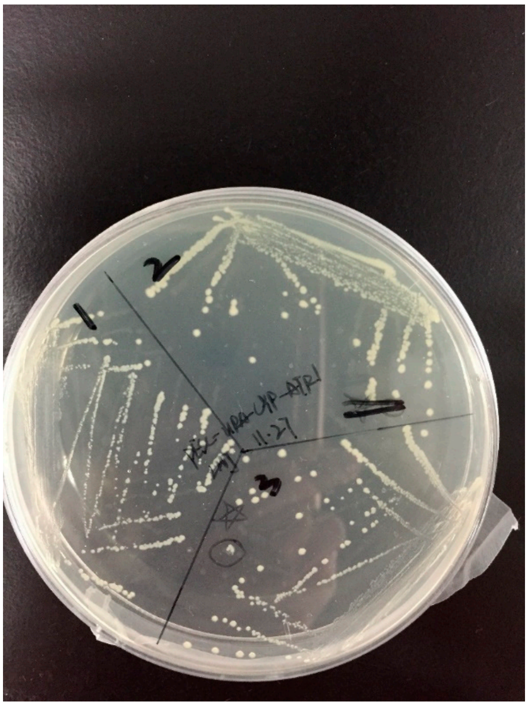

Figure S4. Purification and separation of a pESC-ura-CYP716A12-ATR1 DH5α single colony.

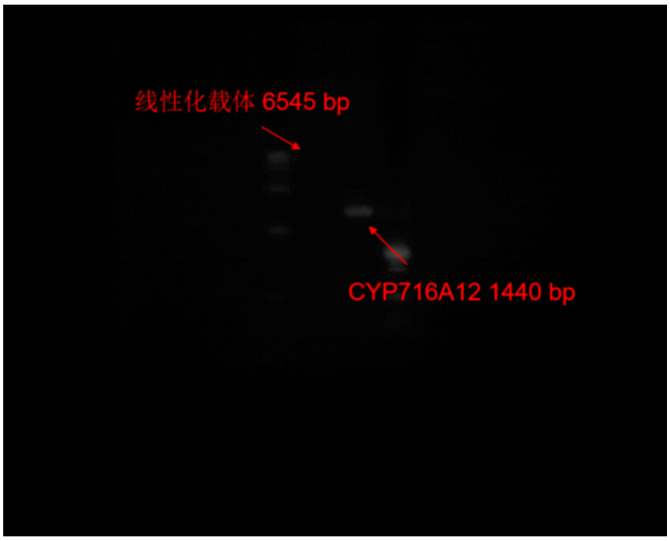

Figure S5. Agarose gel electrophoresis results of the added concentration of vector and targeting gene.

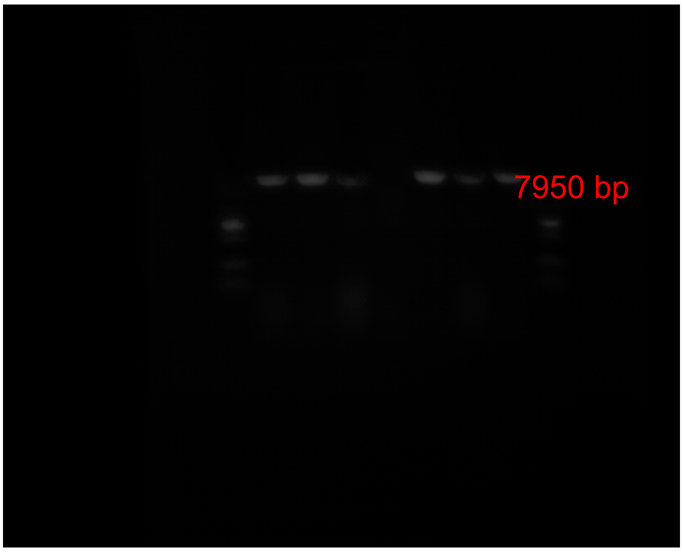

Figure S6. Colony PCR of a pESC-ura-CYP716A12 DH5α single colony.

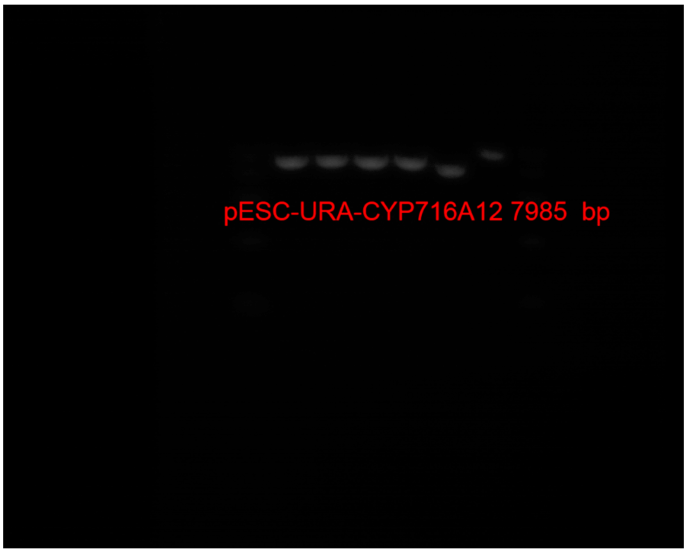

Figure S7. Agarose gel electrophoresis results of plasmid from pESC-ura-CYP716A12 DH5α.

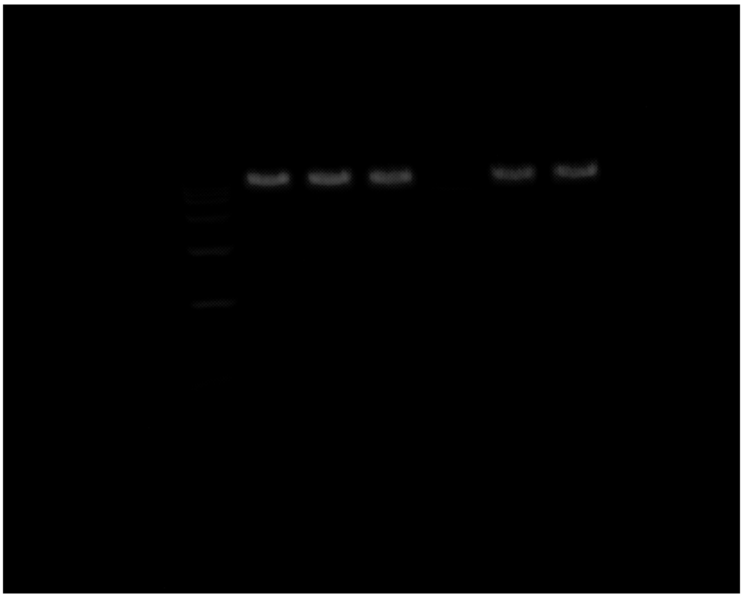

Figure S8. Agarose gel electrophoresis results of linearized pESC-ura-CYP716A12.

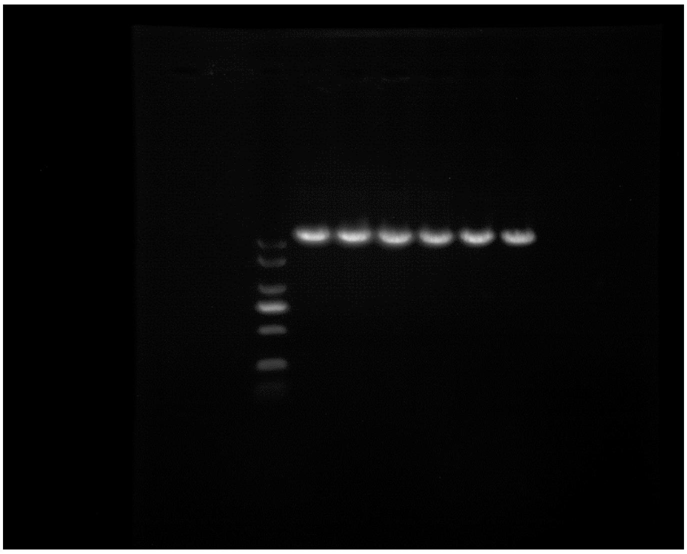

Figure S9. Colony PCR of pESC-ura-CYP716A12-ATR1 DH5α.

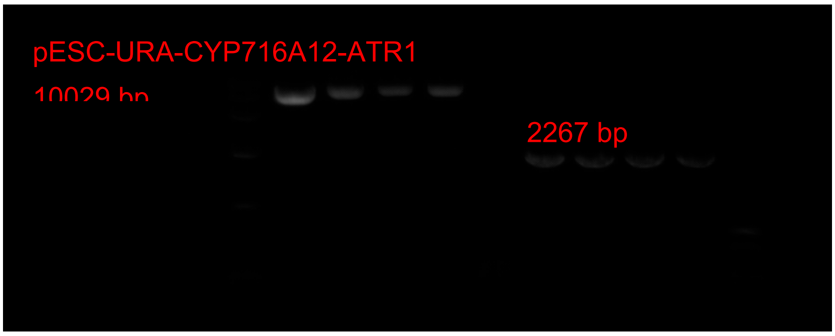

Figure S10. Agarose gel electrophoresis results of plasmid from pESC-ura-CYP716A12-ATR1 DH5α.

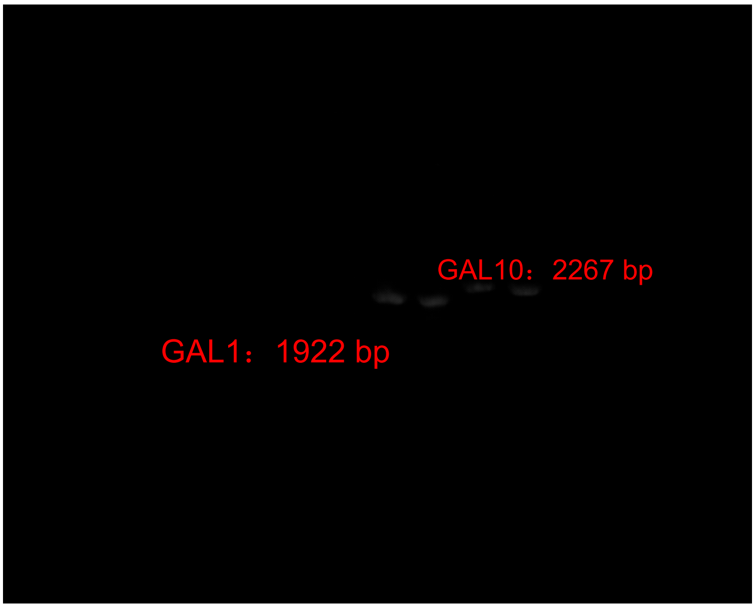

Figure S11. Agarose gel electrophoresis results of specific primer PCR of plasmid from *Saccharomyces cerevisiae* ZJUQH311.

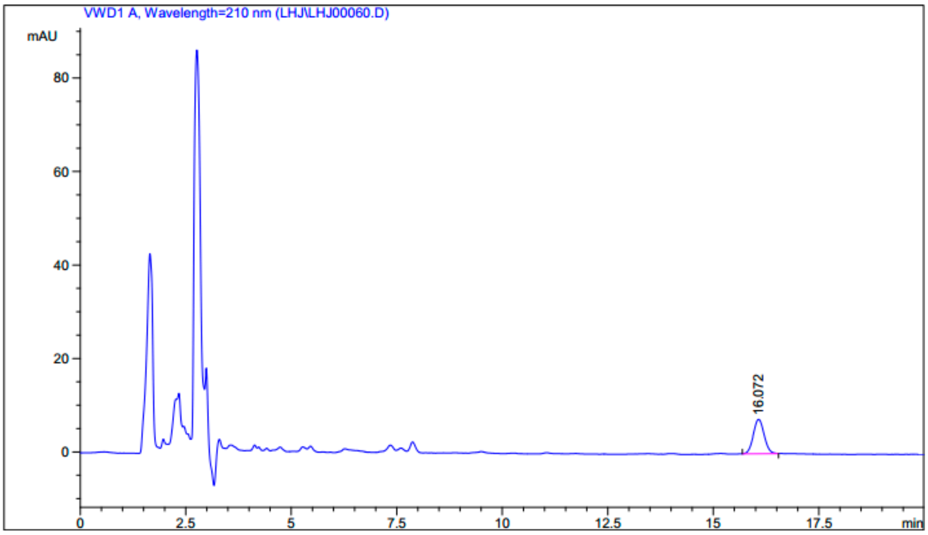

Figure S12. HPLC chromatograms of microsome protein from the total protein extraction.

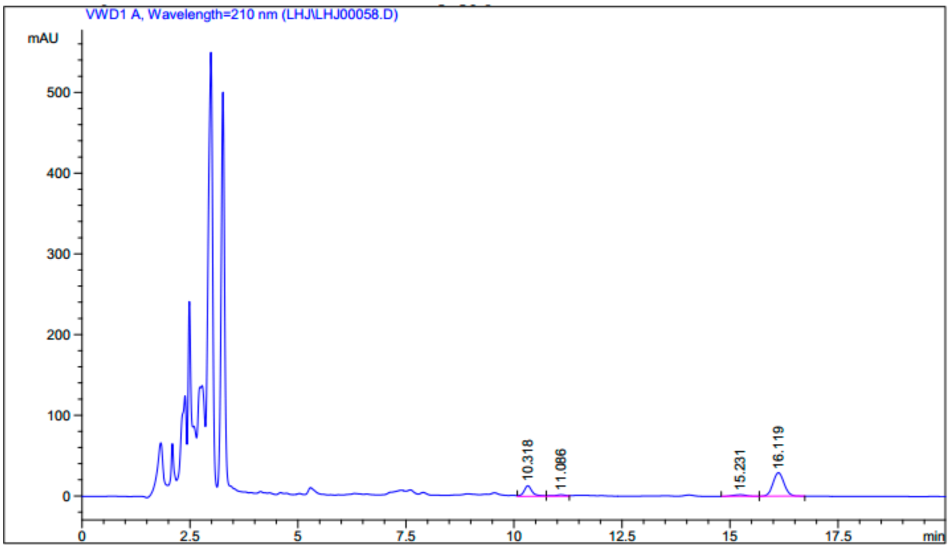

Figure S13. HPLC chromatograms of microsome protein from the microsome protein extraction.

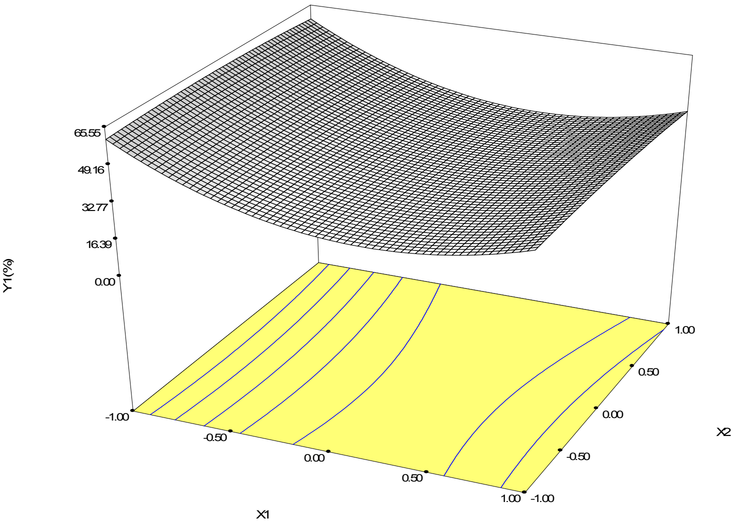

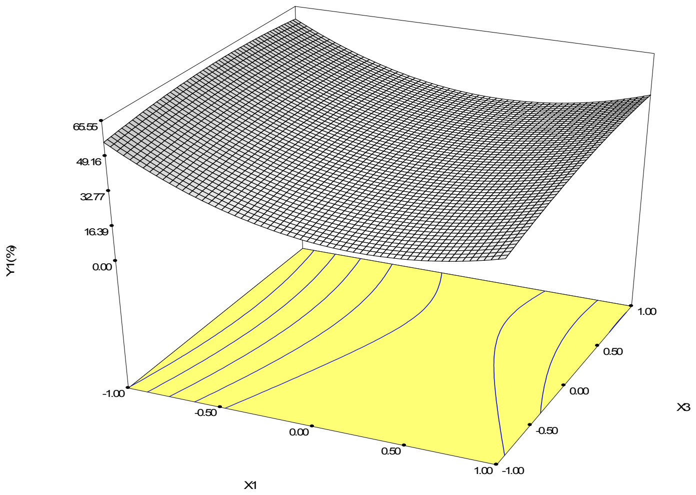

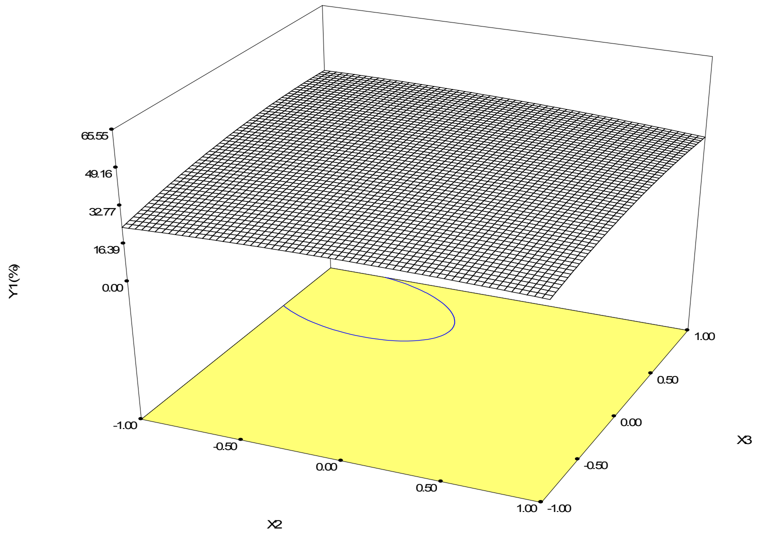

Figure S14. The response surface plot of conversion time (X_1), betulin (X_2), and NADPH (X_3) against betulin conversion.

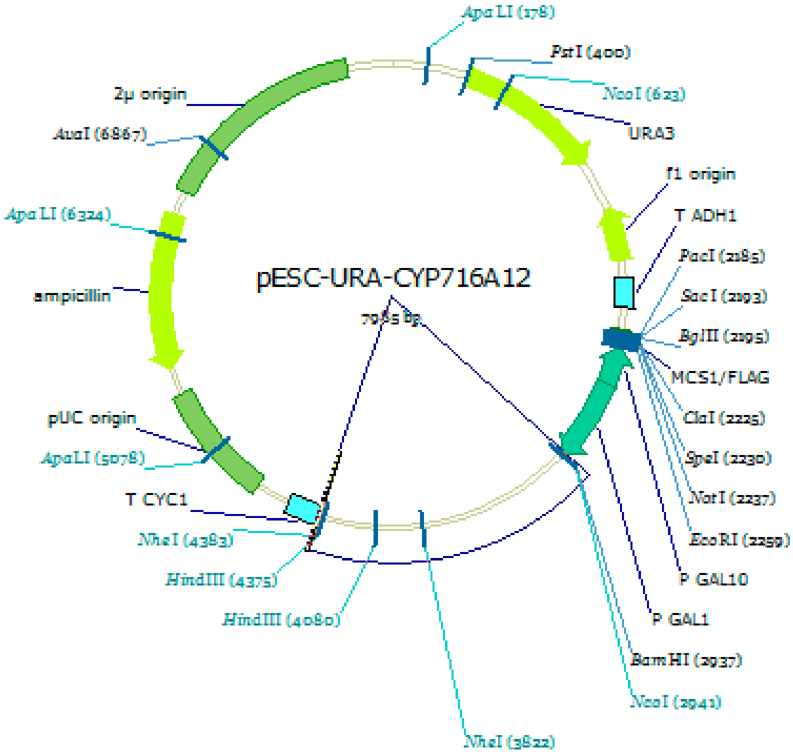

Figure S15. Schematic diagram of pESC-ura-CYP716A12.

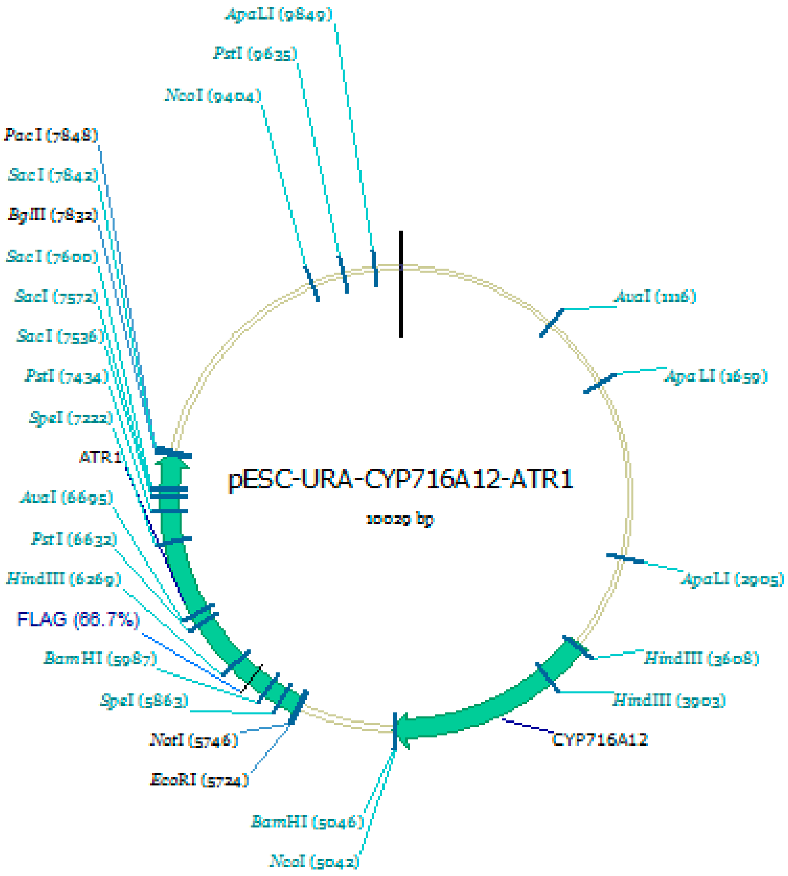

Figure S16. Schematic diagram of pESC-ura-CYP716A12-ATR1.

Table S1. Results of the response surface methodology regression analysis for betulin conversion (Y_1).

Source	Sum of squares	DF	Mean Square	F value	Prob > F
Model	2269.72	9	252.19	9.74	0.0033
X_1	645.31	1	645.31	24.92	0.0016
X_2	11.08	1	11.08	0.43	0.5340
X_3	113.74	1	113.74	4.39	0.0743
X_1^2	1430.38	1	1430.38	55.23	0.0001
X_2^2	13.18	1	13.18	0.51	0.4987
X_3^2	61.40	1	61.40	2.37	0.1675
X_1X_2	1.15	1	1.15	0.04	0.8394
X_1X_3	18.11	1	18.11	0.70	0.4307
X_2X_3	11.82	1	11.82	0.46	0.5210
Residual	181.29	7	25.90		
Lack of Fit	148.86	3	49.62	6.12	0.0563
Pure error	32.43	4	8.11		
Cor Total	2451.01	16			
